# Determinants and reasons for coronavirus disease 2019 vaccine hesitancy in Croatia

**DOI:** 10.3325/cmj.2022.63.89

**Published:** 2022-02

**Authors:** Dragan Bagić, Adrijana Šuljok, Branko Ančić

**Affiliations:** 1 Faculty of Humanities and Social Sciences, University of Zagreb, Zagreb, Croatia; 2 Institute for Social Research in Zagreb, Zagreb, Croatia

## Abstract

**Aim:**

To assess the determinants and reasons for coronavirus disease 2019 (COVID-19) vaccine hesitancy in Croatia.

**Methods:**

The data were collected through a sociological survey by using a mixed-mode approach (computer-assisted web interviewing and computer-assisted telephone interview) on a national sample of 765 adults aged 18 or above. Bivariate (χ^2^ test) and multivariate (binary logistic regression) statistical methods were used.

**Results:**

The rate of COVID-19 vaccine hesitancy was relatively high (35%), with unequal distribution across demographic groups. Binary logistic regression with demographic characteristics as predictors showed that women, younger age groups (especially 25-34-year-olds), persons residing in households with children, inhabitants of smaller settlements, and persons with lower levels of education had higher odds of vaccine hesitancy. Trust in the five main actors responding to the COVID-19 pandemic (the National Civil Protection Headquarters, Government, health care system, scientists-researchers, and media) was also a significant predictor of vaccine hesitancy. Risk perception was an even stronger predictor: persons who perceived SARS-CoV-2 infection as a small risk were more than ten times likelier to be vaccine hesitant than those who perceived it as a great risk.

**Conclusion:**

Social groups that are more prone to vaccine hesitancy need to be approached through different channels and messages by taking into account their trust in institutions and risk perception.

Vaccine hesitancy is defined as a delay in the acceptance or refusal of vaccines despite the availability of vaccine services (1). Numerous studies have tried to explain the main sociological, psychological, and other factors associated with vaccine hesitancy. According to the World Health Organization and the 3C model, vaccine hesitancy is influenced by three main factors: convenience (access to vaccine); confidence (trust in vaccine safety/effectiveness and health care providers/policy-makers); and complacency (health beliefs, perception of disease as low risk) (1). Recent studies across countries have shown that coronavirus disease 2019 (COVID-19) vaccine acceptance was not high (2,3), which makes it difficult to achieve herd immunity and control the spread of the pandemic. Although vaccine-hesitant individuals are a heterogeneous group, these studies have identified that vaccine hesitancy is more prevalent among certain sociodemographic groups (2). No studies so far have assessed the COVID-19 vaccine hesitancy in Croatia. The main aim of this study is to identify the reasons and determinants of COVID-19 vaccine hesitancy in Croatia and identify social groups that might be less willing to get the vaccine.

## Methods

### Respondents and analytical approach

The data were collected between March 4 and April 11, 2021 by using a mixed-mode approach in the second wave of a panel-design study. The first study/wave was conducted in autumn 2020 on a randomized national sample of 1512 respondents, 765 (50.6%) of whom agreed to participate in the follow-up study. Among these 765 respondents, 616 (80.5%) answered a web-based self-completion questionnaire and 149 (19.5%), mostly older and less educated respondents, took part in a computer-assisted telephone interview. The data were weighted to correct deviations of the first sample from the structure of the population of adult private households in Croatia. The data were also weighted based on the propensity for participation in the follow-up study.

After applying the weightings, 52.4% of respondents were female. The average age was 49.18 years (standard deviation 16.16; range 18-88 years). In terms of educational level, 21.3% of respondents had below-secondary education, 56.4% secondary education, and 22.3% tertiary education. A total of 42.7% of respondents were employed, 35.1% were retired, and the rest of the sample had other employment statuses. Respondents were from all size-categories of settlements, with the highest share (38.0%) from the smallest settlement category (up to 2000 inhabitants). Respondents were also from all Croatian regions, with the share of respondents from each of the six regions being approximately proportional to the share of the overall population.

Descriptive statistics are presented, and two models of binary logistic regression were performed with vaccine hesitancy as a binary dependent variable. The first model identified the groups that should be targeted in pro-vaccination campaigns, while the second identified the most effective messengers and messages. The data analysis was performed with SPSS Statistics, version 28.0 (IBM Corp, Armonk, NY, USA).

### The outcome variable

Similar to other studies (4), vaccination hesitancy was measured with the following simple question about the intention to receive the COVID-19 vaccine: “How likely are you to get vaccinated against COVID-19 when the vaccine becomes available for your group?” Six answer options were provided: “I have already been vaccinated” (code 0); “I will definitely get vaccinated” (code 1); “I will likely get vaccinated” (code 2); “I will likely not get vaccinated” (code 3); and “I will definitely not get vaccinated” (code 4). The outcome variable was recoded into a binary variable: 0, 1, and 2 representing the non-hesitant group and 3 and 4 representing the hesitant group (coded 1). Seven respondents who had not answered the main outcome question were excluded from the analysis.

Two secondary outcome variables representing the reasons for vaccine acceptance and hesitation were also included. Respondents who said they were likely to get vaccinated were offered five reasons with answers on a four-point scale ranging from 1 (“It completely applies to me”) to 4 (“It does not apply to me at all”). Respondents who were hesitant about getting vaccinated were offered five reasons with answers on the same four-point scale.

### Independent variables

In the first regression model, six variables were used as predictors of COVID-19 vaccine hesitancy: sex; age group; education level; employment status (coded in a binary form as 1 for those employed); material deprivation scale (standard EU indicator adopted in 2009 by 27 EU member states and EC, defined as the ability of a household to afford nine types of services and goods) as a proxy for income level (coded in a binary form, with code 1 for those with values 3 and higher); settlement size; and region.

The second model added an attitudinal variable measuring trust in the five main actors responding to the COVID-19 pandemic: the National Civil Protection Headquarters; the Government; the health care system; scientists-researchers; and the media. The level of trust in each actor was expressed on a four-point scale ranging from 1 (“I have full trust”) to 4 (“I have no trust at all”). Two additional variables measured whether respondents had already recovered from COVID-19 and how they perceived the risk of SARS-CoV-2 infection, by using a three-point scale ranging from 1 (“Small or no risk”) to 3 (“Extreme risk”).

Missing values are excluded listwise. In total, 21 respondents were excluded from the analysis because of a missing value in at least one variable used in the final regression model, with 744 respondents remaining.

## Results

Overall, 11.5% of respondents had already been vaccinated against COVID-19 with the first or second dose (1), and further 52.4% had the intention to get vaccinated (definitely or likely). On the other hand, 35.3% of respondents were not willing to get vaccinated, among which 47% responded that they will definitely not get vaccinated (Table 1).

**Table 1:** The responses to the question “How likely are you to get vaccinated against COVID-19 when the vaccine becomes available for your group?” in a national Croatian sample of 765 adults

Question	%
I have already been vaccinated	11.5
I will definitely get vaccinated	27.0
I will likely get vaccinated	25.4
I will likely not get vaccinated	18.7
I will definitely not get vaccinated	16.6
No answer	0.7

Among the respondents who had been vaccinated or who intended to get vaccinated, the predominant reasons for vaccination were to protect others from being infected (95%), trust in vaccine effectiveness and safety (86%), and the belief that the vaccine enables us to return to normal life (83%) (Table 2). The smallest number of respondents mentioned self-assessed membership of at-risk groups and being frequently in high-risk situations.

**Table 2:** Reasons for getting vaccinated in a national Croatian sample of 765 adults (results are expressed as percentages)

Reason	It does not apply to me at all	It mostly does not apply to me	It mostly applies to me	It completely applies to me	No answer
I believe I belong to an at-risk group	27.5	18.7	19.6	33.6	0.6
I have trust in the vaccine’s effectiveness and safety	2.9	9.8	47.6	38.2	1.5
I believe that the vaccine will enable me to return to a normal life	2.6	10.3	45.1	38.1	3.8
I want to protect others from infection	1.0	2.4	24.6	70.5	1.7
I believe I am frequently at risk of infection	13.3	36.2	24.0	25.3	1.1

The predominant reasons for vaccine hesitancy were the belief that the COVID-19 vaccines were not sufficiently safe (82%), the belief that only naturally acquired immunity offers true protection (72%), and distrust in vaccines in general (71%) (Table 3). However, about two-thirds of respondents offered other reasons for hesitancy: a distrust in vaccine effectiveness and the assessment that the virus was not dangerous for the respondent. Women more often than men perceived that COVID-19 vaccines were not sufficiently safe (87% vs 75.3%).

**Table 3:** Reasons for vaccine hesitancy in a national Croatian sample of 765 adults (results are expressed as percentages)

Reason	It does not apply to me at all	It mostly does not apply to me	It mostly applies to me	It completely applies to me	No answer
I believe the virus is not dangerous for me	10.8	22.5	45.3	21.1	0.2
I do not believe that the vaccine is sufficiently effective in protecting me from the virus	11.7	19.1	36.9	29.7	2.6
I believe that only natural immunity, acquired by recovering from the disease, can truly protect me	8.0	18.6	42.1	29.8	1.5
I do not believe that the vaccines offered to us are sufficiently safe	6.6	9.3	37.5	44.7	2.0
I am skeptical toward vaccination in general	14.2	14.3	30.0	41.4	0.1

### Bivariate analysis

Women were slightly more hesitant than men to receive the COVID-19 vaccine (39.7% vs 31.0%). Younger respondents were significantly more hesitant than older ones, especially when we compared the 25-34 and the 55-64 age groups (66.6% vs 19.0%) (Table 4). Differences by the level of education and level of material deprivation were not significant. The share of hesitant respondents decreased as the settlement size increased, with 42.2% of inhabitants of small settlements being vaccine hesitant compared with 29.9% in cities with more than 80 000 inhabitants. Differences between regions were significant at the bivariate level, with the smallest share of the vaccine hesitant respondents being in Istria, Primorje, and Gorski Kotar (20.6%), and the highest being in Central (45.3%) and Northern Croatia (44.4%). A higher share of hesitant respondents lived in households with children (47.3%) compared with households without children (30.2%).

**Table 4:** Vaccine acceptance/hesitancy according to respondents’ characteristics

		Unweighted n	Already vaccinated, likely or definitely will get vaccinated (%)	Likely or definitely will not get vaccinated (%)	χ^2^ test
Entire sample		758	64.4	35.6	-
Sex	male	317	69.0	31.0	χ^2^ = 6.214 df = 1 *P* = 0.013
	female	441	60.3	39.7	
Age	18-24	43	51.2	48.8	χ^2^ = 71.939 df = 5 *P* < 0.001
	25-34	85	33.4	66.6	
	35-44	154	63.6	36.4	
	45-54	176	56.9	43.1	
	55-64	174	81.0	19.0	
	65+	126	76.0	24.0	
Education level	no secondary school	43	61.9	38.1	χ^2^ = 4.644 df = 4 *P* = 0.326
	three-year vocational secondary school	50	59.1	40.9	
	technical or grammar secondary school	321	64.1	35.9	
	bachelor’s degree	144	65.1	34.9	
	master's degree or higher	200	73.5	26.5	
Material deprivation	not materially deprived	618	65.1	34.9	χ^2^ = 0.518 df = 1 *P* = 0.472
	materially deprived	140	62.1	37.9	
Settlement size	up to 2000	243	57.8	42.2	χ^2^ = 10.207 df = 3 *P* = 0.017
	2001 to 10 000	109	64.9	35.1	
	10 001 to 80 000	172	68.9	31.1	
	80 001+	234	71.0	29.0	
Region	Slavonia	139	60.6	39.4	χ^2^ = 17.069 df = 5 *P* = 0.004
	Central Croatia	95	54.7	45.3	
	Northern Croatia	101	55.6	44.4	
	Zagreb	214	65.8	34.2	
	Istria, Primorje, and Gorski Kotar	81	79.4	20.6	
	Dalmatia	128	66.8	33.2	
Children in the household (0-17)	no	499	69.8	30.2	χ^2^ = 21.107 df = 1 *P* < 0.001
	yes	259	52.6	47.4	
Trust in the National Civil Protection Headquarters	no	327	52.9	47.1	χ^2^ = 40.691 df = 1 *P* < 0.001
	yes	429	75.2	24.8	
Trust in scientists-researchers	no	126	32.4	67.6	χ^2^ = 87.528 df = 1 *P* < 0.001
	yes	629	72.8	27.2	
Trust in the media	no	408	54.0	46.0	χ^2^ = 45.339 df = 1 *P* < 0.001
	yes	347	77.8	22.2	
Trust in the Government	no	442	59.6	40.4	χ^2^ = 15.039 df = 1 *P* < 0.001
	yes	310	73.6	26.4	
Trust in the health care system	no	231	48.0	52.0	χ^2^ = 47.185 df = 1 *P* < 0.001
	yes	526	73.0	27.0	
Disease or symptoms of COVID-19	no	640	63.3	36.7	χ^2^ = 2.660 df = 1 *P* = 0.103
	yes	118	72.1	27.9	
Assessment of SARS-CoV-2 virus infection risk	small or no risk	177	25.6	74.4	χ^2^ = 195.345 df = 12 *P* < 0.001
	significant risk	389	76.5	23.5	
	extreme risk	192	84.5	15.5	

In general, respondents who expressed trust in the five main actors responding to the pandemic in Croatia were less vaccine hesitant, that is, a higher percentage of those who trusted these actors was willing to get vaccinated. The biggest difference in the share of vaccine-hesitant respondents related to trust in scientists-researchers: those who distrusted scientists-researchers were 2.48 times more likely (67.6% vs 27.2%) to express hesitancy than those who trusted them. Distrust in the media resulted in a 2-fold increase in the likelihood of hesitancy (46.0% vs 22.2%). Similarly, lack of trust in the National Civil Protection Headquarters and the health care system resulted in respondents being 1.9 times more likely to be vaccine hesitant. Distrust in the Government was least likely to increase the likelihood of hesitancy, with those who distrusted the Government being only 1.5 more likely to be hesitant (40.4% vs 26.4%).

The biggest differences in vaccine acceptance were related to the perceived risk of getting infected with the SARS-CoV-2 virus. The vast majority (almost two-thirds) of respondents who perceived the risk as being small or non-existent were hesitant or refused to receive the vaccine. Respondents who perceived the risk as being significant were mostly willing to get vaccinated; however, about a quarter of them were still hesitant or refused to do so, while the share of such respondents was even lower among those who perceived the risk as being great.

Among respondents with a confirmed or diagnosed SARS-CoV-2 infection based on symptoms, the difference between those who were hesitant or willing to be vaccinated was not significant (36.7% vs 27.9%).

## Multivariate analysis

In the first regression model, comprised of demographic characteristics, sex was a significant predictor of vaccine hesitancy, with women being 1.7 times more to be hesitant, all other characteristics being equal. All age categories, except the 35-44 age group, were more likely to be hesitant compared with the oldest age group, with respondents aged 25-34 being seven times more likely to be hesitant than respondents from the oldest age group, all other characteristics being equal. Respondents with lower levels of education were more likely to be hesitant compared with those with a master’s degree, with respondents who had not completed secondary school or who had completed three-year vocational secondary school being about three times more likely to be hesitant than respondents with a master’s degree, all other characteristics being equal. Respondents from the two smallest settlement categories (up to 2000 and between 2001-10 000 inhabitants) were more likely to be hesitant than respondents from the largest cities. Respondents living with children in a household were 1.4 times more likely to be vaccine hesitant, all other characteristics being equal. By knowing a person’s demographic characteristics, it was possible to precisely predict the vaccination intention for about 71% of respondents, with the likelihood of predicting vaccine hesitancy being 43.6% and the likelihood of predicting vaccine acceptance being 86.6%.

In the second model, variables measuring trust in the five main actors responding to the pandemic were added to demographic characteristics, together with the two COVID-related variables (disease or symptoms of COVID-19 and perception of the risk of infection). Trust in all five actors was a significant predictor of the likelihood of vaccine hesitancy, controlling for respondents’ demographic characteristics. All other characteristics being equal, respondents who trusted the National Civil Protection Headquarters, scientists-researchers, and media were about twice less likely to be vaccine hesitant than those who distrusted them (odds ratios: 2.0, 2.1, and 2.0, respectively). Those who trusted the health care system were about 1.6 times less likely to be vaccine hesitant. On the other hand, respondents who trusted the Government were 2.5 times more likely to be vaccine hesitant. All other characteristics being equal, respondents whose COVID-19 infection was confirmed by testing or diagnosed by symptoms were 2.2 times less likely to be hesitant. The most striking finding was that those who perceived the risk of infection as small or non-existent were 10.5 times more likely to be hesitant than those who perceived the risk of infection as extreme.

In the second model, when additional variables were introduced, the indicators of the demographic set of variables changed somewhat. The groups that still had a higher odds ratio of hesitancy in comparison with reference groups were women, 25-34-year-olds, persons with lower levels of education, and members of households with children (Table 5). However, differences with regards to settlement size were no longer significant, whereas material deprivation became a significant predictor (respondents from materially deprived households were 1.6 times more likely to be vaccine hesitant).

**Table 5:** Regression models assessing the likelihood of vaccination

		Model 1				Model 2			
		odds ratio	95% confidence interval		p	odds ratio	95% confidence interval		P
Constant		0.042			<0.001	0.12			0.003
Sex	male	1				1			
	female	1.715	1.215	2.422	<0.001	1.662	1.096	2.519	0.017
Age	18-24	3.349	1.723	6.511	<0.001	1.202	0.53	2.726	0.660
	25-34	7.052	3.583	13.88	<0.001	3.191	1.414	7.198	0.005
	35-44	1.604	0.874	2.942	0.127	0.673	0.32	1.417	0.297
	45-54	2.267	1.262	4.072	0.006	1.353	0.669	2.736	0.400
	55-64	0.523	0.285	0.962	0.037	0.524	0.262	1.048	0.068
	65+	1				1			
Education	no secondary school	3.329	1.685	6.578	<0.001	2.301	0.989	5.355	0.053
	three-year vocational secondary school	3.201	1.515	6.764	0.002	3.118	1.288	7.549	0.012
	four-year secondary school or grammar secondary school	1.864	1.038	3.347	0.037	1.973	0.991	3.927	0.053
	bachelor’s degree	1.983	0.936	4.2	0.074	2.808	1.189	6.633	0.019
	master's degree or higher	1				1			
Material deprivation	not materially deprived	1				1			
	materially deprived	1.181	0.783	1.781	0.428	1.565	0.973	2.518	0.065
Settlement size	up to 2,00	1.711	1.021	2.867	0.041	1.109	0.605	2.035	0.738
	2001 to 10 000	1.933	1.078	3.468	0.027	1.646	0.834	3.249	0.151
	10 001 to 80 000	1.258	0.713	2.221	0.429	1.232	0.636	2.387	0.536
	80 001+	1				1			
Region	Slavonia	0.994	0.578	1.711	0.984	0.843	0.448	1.586	0.596
	Central Croatia	0.821	0.421	1.6	0.563	0.740	0.333	1.643	0.459
	Northern Croatia	1.289	0.68	2.445	0.437	1.201	0.553	2.609	0.643
	Zagreb	1.096	0.646	1.86	0.734	0.944	0.496	1.795	0.860
	Istria, Primorje, and Gorski Kotar	0.576	0.298	1.115	0.102	0.570	0.263	1.237	0.155
	Dalmatia	1				1			
Children in the household (0-17)	no	1				1			
	yes	1.401	0.94	2.087	0.097	1.962	1.223	3.15	0.005
Trust in the National Civil Protection Headquarters	no					1			
	yes					0.508	0.284	0.909	0.022
Trust in scientists-researchers	no					1			
	yes					0.470	0.273	0.808	0.006
Trust in the media	no					1			
	yes					0.508	0.327	0.789	0.003
Trust in the Government	no					1			
	yes					2.500	1.366	4.575	0.003
Trust in the health care system	no					1			
	yes					0.633	0.381	1.051	0.077
Disease or symptoms of COVID-19	no					1			
	yes					0.452	0.236	0.864	0.016
Assessment of SARS-CoV-2 virus infection risk	small or no risk					10.517	5.705	19.388	<0.001
	significant risk					1.463	0.854	2.507	0.166
	extreme risk					1			

The model with all the observed variables had a predictivity of 80.8%, with 90.2% precision in predicting vaccine acceptance and 64.2% precision in predicting vaccine hesitancy.

## Discussion

In the early spring of 2021, when this research was finalized, around 16% of the adult population in Croatia received at least one dose of the COVID-19 vaccine. In this study, the share of the adult population that was vaccine hesitant was relatively high: around 18.7% of the population was reluctant to be vaccinated (“likely not”) and 16.7% was determined not to get vaccinated (“definitely not”). Among these groups, vaccine hesitancy was mostly a matter of distrust regarding the safety and effectiveness of the COVID-19 vaccines. Similar findings regarding fears about side effects and future negative effects of vaccines have been noted in many previous studies, eg, in recent studies from the UK (4) and Croatia (5). However, a large proportion of our respondents also perceived vaccination against COVID-19 as unnecessary due to the harmless nature of the SARS-CoV-2 virus and to a preference for natural immunity. The latter could be more difficult to influence by a campaign based solely on emphasizing vaccine safety and outweighing the benefits over the risks, because these respondents do not perceive any significant risk.

The analysis confirmed that vaccine hesitancy was more prevalent among the following demographic groups, as confirmed in many similar studies (2,4,6-8): young people, women, persons with lower levels of education, members of households with children, and inhabitants of smaller settlements. These demographic groups should be targeted in a vaccination campaign. Vaccine hesitation is especially prevalent in the 25-34 age group. Previous research has shown that the younger population in Croatia more often expresses negative attitudes about facemasks, exhibits lower levels of protective behavior, has a lower risk perception regarding SARS-CoV-2 infection, and has a lower level of trust in the institutions responding to the pandemic (9,10). Obviously, a special approach is needed in addressing this group because they are less prone to protective measures and are more vaccine hesitant. Women were also more prone to hesitation, although they were more often the targets of public health campaigns because of their greater compliance with public health directives and the caregiver role in the family (11). Researchers attribute women’s higher hesitancy rate to multiple reasons, including a lower propensity to take risks than men. In our study, women perceived more often than men that vaccine was not sufficiently safe. Misinformation about the effect of the COVID-19 vaccine on fertility, as well as other risks (very rare cases of thrombosis after vaccination), can be some of the drivers of vaccine hesitancy among women.

The importance of public trust and credibility of actors (political institutions, the health care system, scientists) involved in vaccination strategy/policy was indicated previously (12-14). A study in UK (14) found that groups that were willing to be vaccinated were more likely to trust the government’s managing of the pandemic. According to our findings, (dis)trust in institutions that respond to the pandemic was also a significant factor in vaccine hesitation, which is an essential element to consider when choosing dissemination channels and actors in communication campaigns. Although distrust in all institutions increased the likelihood of hesitation, the impact of distrust in scientists was particularly pronounced. As many as two-thirds of respondents who did not trust scientists were vaccine hesitant and those who distrusted scientists were more than twice as likely to be vaccine hesitant. This clearly shows that the underlying causes of hesitancy are distrust in scientific studies and scientific arguments related to the effectiveness and safety of vaccines. The conclusion is that *ad hoc* educational campaigns may not be an effective way of reducing vaccine hesitation and increasing vaccine uptake. Other types of arguments for vaccine acceptance should be sought – and indeed, other types of communicators. For example, whereas vaccine hesitant groups were more likely to distrust the National Civil Protection Headquarters, the health care system, scientists-researchers, and the media, they were more likely to trust the Government. This finding can be explained by the fact that there are certain political factors that link trust in the Government and attitudes toward vaccination among some respondents. This has the implication that the Government, or the main ruling party, could disseminate pro-vaccination messages more effectively to vaccine hesitant groups through political channels.

The strongest factor of hesitation in our study was perceived low danger of COVID-19 infection. Previous studies have found that perceiving COVID-19 infection as a higher risk/danger was associated with a higher acceptance of the COVID-19 vaccine (14). The recent rise in COVID-19 infections and deaths in Croatia (in October and November 2021) has been accompanied by an increase in the vaccine uptake (which can be interpreted as perception of higher threat by individuals), as well as by the introduction of obligatory COVID certificates for some professions. This points to the conclusion that previous public communication about the dangers and the course of the pandemic has failed, since a significant part of the population refused to accept the severity of the disease and the broader effects of the pandemic. Namely, there have been dissonant tones between different actors in the media and in the public about the suitability of epidemiological measures and the need for vaccination. Public discourse also primarily emphasized certain groups as being vulnerable (older people and persons with existing health conditions). This may have contributed to the failure of previous public communication by constructing a low risk perception in certain social groups, as already noticed in studies (14,15). Additionally, the narrative of social solidarity (“Think of others, get vaccinated”, the official slogan of Croatian pro-vaccination campaign) was not successful among certain social groups. Of course, the asymmetric risk of illness with regards to health and age has played a role, but also the level of social solidarity among certain groups decreased as the pandemic lasted. A communication strategy explaining the risks and dangers of the COVID-19 pandemic therefore requires a nuanced approach that applies different arguments depending on the age, sex, and education level of the target groups.

This study has some limitations in terms of the generalizability of its findings. The study sample, although being national and representative for the Croatian adult population, is not completely random. The article was based on data collected in the second wave of a panel survey, with significant self-selection effect. In addition, attitudes on vaccination can significantly change over time under the influence of changes in context (eg, new pandemic waves, changes in public and media discourse, new information about the safety and effectiveness of vaccines).

In conclusion, this study reveals several practical implications for public health policy. Our findings are coherent with previous studies showing the link between COVID-19 vaccine hesitancy and age, sex, education, urban/rural residence, ethnicity, as well as the link between vaccine hesitancy and trust in institutions and, especially, COVID-19 risk perception.

The findings suggest that vaccination campaigns should be strategically focused on specific sociodemographic groups that are more likely to be vaccine hesitant. They should not only be conceived as educational campaigns, or be focused on scientific arguments about safety and effectiveness of vaccine, and on the authority of science and scientists, since vaccine-hesitant groups do not trust scientists and experts. Additionally, the study findings suggest that the previous approach to the public communication of COVID-19 risks in Croatia should be revised to exclude dissonant tones about the severity of disease in the media and public.

## References

[1] MacDonald NE SAGE working group on vaccine hesitancy. vaccine hesitancy: definition, scope and determinants. Vaccine 2015 33 4161 4 10.1016/j.vaccine.2015.04.036 25896383

[2] Troiano G Nardi A Vaccine hesitancy in the era of COVID-19. Public Health 2021 194 245 51 10.1016/j.puhe.2021.02.025 33965796 PMC7931735

[3] Sallam M COVID-19 vaccine hesitancy worldwide: a concise systematic review of vaccine acceptance rates. Vaccines (Basel) 2021 9 160 10.3390/vaccines9020160 33669441 PMC7920465

[4] Robertson E Reeve KS Niedzwiedz CL Moore J Blake M Green M Predictors of COVID-19 vaccine hesitancy in the UK household longitudinal study. Brain Behav Immun 2021 94 41 50 10.1016/j.bbi.2021.03.008 33713824 PMC7946541

[5] Miškulin M, Gavranić K, Domaćinović T, Pavlović N, Vukoja I, Miškulin I. Antivaccination attitudes and intention to take COVID-19 vaccine in Croatian adult population. Eur J Public Health. Oct 31(3).

[6] Ward JK Alleaume C Peretti-Watel P COCONEL Group. The French public’s attitudes to a future COVID-19 vaccine: The politicization of a public health issue. Soc Sci Med 2020 265 113414 10.1016/j.socscimed.2020.113414 33038683 PMC7537647

[7] Soares P Rocha JV Moniz M Gama A Laires PA Pedro AR Factors associated with COVID-19 vaccine hesitancy. Vaccines (Basel) 2021 9 300 10.3390/vaccines9030300 33810131 PMC8004673

[8] Schernhammer E Weitzer J Laubichler MD Birmann BM Bertau M Zenk L Correlates of COVID-19 vaccine hesitancy in Austria: trust and the government J Public Health (Oxf) 2021May 5:fdab122 10.1093/pubmed/fdab122 33948665 PMC8135852

[9] Bagić D Šuljok A **„**Stavi masku i odmakni se“ – istraživanje determinanti protektivnog ponašanja od bolesti COVID-19 u Hrvatskoj [in Croatian] Soc Prost. 2021 219 119 47

[10] Ančić B Cepić D Tko su antimaskeri u Hrvatskoj? Prilog istraživanju antimaskerske reakcije tijekom pandemije bolesti COVID-19 u Hrvatskoj [in Croatian] Soc Prost. 2021 219 187 21

[11] Doyal L Sex, gender, and health: the need for a new approach. BMJ 2001 323 1061 3 10.1136/bmj.323.7320.1061 11691769 PMC1121552

[12] Yaqub O Castle-Clarke S Sevdalis N Chataway J Attitudes to vaccination: a critical review. Soc Sci Med 2014 112 1 11 10.1016/j.socscimed.2014.04.018 24788111

[13] Larson HJ Schulz WS Tucker JD Smith DM Measuring vaccine confidence: introducing a global vaccine confidence index PLoS Curr 2015Feb 25;7:ecurrents.outbreaks.ce0f6177bc97332602a8e3fe7d7f7cc4 10.1371/currents.outbreaks.ce0f6177bc97332602a8e3fe7d7f7cc4 25789200 PMC4353663

[14] Jennings W Stoker G Bunting H Valgarðsson VO Gaskell J Devine D Lack of trust, conspiracy beliefs, and social media use predict COVID-19 vaccine hesitancy. Vaccines (Basel) 2021 9 593 10.3390/vaccines9060593 34204971 PMC8226842

[15] Seale H Heywood AE McLaws ML Ward KF Lowbridge CP Van D Why do I need it? I am not at risk! Public perceptions towards the pandemic (H1N1) 2009 vaccine. BMC Infect Dis 2010 10 99 10.1186/1471-2334-10-99 20403201 PMC2864274

